# Investigating the effectiveness of web‐based HIV self‐test distribution and linkage to HIV treatment and PrEP among groups at elevated risk of HIV in Viet Nam provinces: a mixed‐methods analysis of implementation from pilot to scale‐up

**DOI:** 10.1002/jia2.26264

**Published:** 2024-07-05

**Authors:** Van Thi Thuy Nguyen, Yasmin Dunkley, Vo Hai Son, Augustine T. Choko, Phan Thi Thu Huong, Pham Duc Manh, Truong Minh Truong, Huynh Minh Truc, Dap Thanh Giang, Le Thanh Tung, Van Dinh Hoa, Rachel Baggaley, Cheryl Johnson

**Affiliations:** ^1^ World Health Organization Country Office in Viet Nam Hanoi Viet Nam; ^2^ London School of Hygiene and Tropical Medicine London UK; ^3^ Viet Nam Administration for HIV/AIDS Prevention and Control Ministry of Health Hanoi Viet Nam; ^4^ Malawi Liverpool Wellcome Trust Clinical Research Programme Blantyre Malawi; ^5^ Department of International Health Liverpool School of Tropical Medicine Liverpool UK; ^6^ The Global Fund supported project on HIV/AIDS Hanoi Viet Nam; ^7^ Can Tho Centre for Disease Control Can Tho city Viet Nam; ^8^ Minh Phat Social Enterprise for Community development Nghe An Viet Nam; ^9^ Hanoi Medical University Hanoi Viet Nam; ^10^ Global HIV Hepatitis and STI Programmes World Health Organization Geneva Switzerland

**Keywords:** HIV, key populations, online distribution, self‐testing, Viet Nam, virtual intervention

## Abstract

**Introduction:**

In Viet Nam, key populations (KPs) face barriers accessing HIV services. Virtual platforms can be leveraged to increase access for KPs, including for HIV self‐testing (HIVST). This study compares reach and effectiveness of a web‐based HIVST intervention from pilot to scale‐up in Viet Nam.

**Methods:**

A mixed‐methods explanatory sequential design used cross‐sectional and thematic analysis. The pilot launched in Can Tho in November 2020, followed by Hanoi and Nghe An in April 2021. Scale‐up included Can Tho and Nghe An, with 21 novel provinces from April to December 2022.

After risk assessment, participants registered on the website, receiving HIVST (OraQuick®) by courier, peer educator or self‐pick‐up. Test result reporting and completing satisfaction surveys were encouraged.

Intervention reach was measured through numbers accessing the testing, disaggregated by demographics, and proportion of individuals reporting self‐testing post‐registration. Effectiveness was measured through numbers reporting self‐test results, testing positive and linking to care, and testing negative and using HIVST to manage pre‐exposure prophylaxis (PrEP) use. Thematic content analysis of free‐text responses from the satisfaction survey synthesized quantitative outcomes.

**Results:**

In total, 17,589 participants registered on the HIVST website; 11,332 individuals ordered 13,334 tests. Participants were generally young, aged <25 years (4309/11,332, 38.0%), male (9418/11,332, 83.1%) and men who have sex with men (6437/11,332, 56.8%). Nearly half were first‐time testers (5069/11,332, 44.9%). Scale‐up participants were two times more likely to be assigned female at birth (scale‐up; 1595/8436, 18.9% compared to pilot; 392/3727, 10.5%, *p* < 0.001). Fewer test results were reported in scale‐up compared with pilot (pilot: 3129/4140, 75.6%, scale‐up: 5811/9194, 63.2%, *p* < 0.001).

6.3% of all tests were reactive (pilot: 176/3129, 5.6% reactive compared to scale‐up: 385/5811, 6.6% reactive, *p* = 0.063); of which most linked to care (509/522, 97.5%). One‐fifth of participants with a negative test initiated or continued PrEP (pilot; 19.8%, scale‐up; 18.5%, *p* = 0.124). Thematic analysis suggested that community delivery models increased programmatic reach. Live chat may also be a suitable proxy for staff support to increase result reporting.

**Conclusions:**

Web‐based self‐testing in Viet Nam reached people at elevated risk of HIV, facilitating uptake of anti‐retroviral treatment and direct linkage to PrEP initiations. Further innovations such as the use of social‐network testing services and incorporating features powered by artificial intelligence could increase the effectiveness and efficiency of the approach.

## 1 INTRODUCTION

In December 2021, an estimated 230,000 people were living with HIV in Viet Nam. General population HIV prevalence was less than 0.3%, with the epidemic concentrated in three key populations (KPs); people who inject drugs (PWID, 12.7%), men who have sex with men (MSM, 13.3%) and female sex workers (FSWs, 3.1%) [1]. Transgender women and amphetamine‐type stimulant (ATS) users also face an elevated risk of HIV in Viet Nam [2]. Viet Nam has a well‐established HIV prevention programme targeting KPs, including an essential package of HIV prevention services delivered through community‐based organizations [2]. This includes pre‐exposure prophylaxis (PrEP) provision for KPs; seeking to increase PrEP use five‐fold from 10,005 KPs in June 2020 to 55,000 individuals by the end of 2023 [3].

However, KPs continue to face challenges accessing HIV services in Viet Nam, because of stigma, discrimination, limited availability of services and structural challenges such as the criminalization of sex work and injecting drug use. Qualitative research in Viet Nam has demonstrated how the COVID‐19 pandemic exacerbated access challenges faced by KP; public health services were suspended to comply with social distancing orders, with HIV programming removed as a national priority in 2021. Access to and coverage of HIV services was reduced and KPs faced increased financial risks through reductions in government subsidies of social health insurance premiums for HIV services [4].

Virtual interventions are now recommended by the World Health Organization (WHO) to reach KPs with HIV testing [5, 6, 7]. Platforms in many countries have integrated HIV self‐testing (HIVST) models into a digital or web‐assisted service—with various formats of digital service model increasing testing uptake and coverage compared to facility‐only models. Digital platforms have been demonstrated as preferable in some settings by first‐time testers, and able to link individuals to care [8, 9, 10, 11]. HIVST has also been shown to be potentially preferred for individuals accessing PrEP [12, 13]. WHO currently encourages that those taking PrEP test quarterly; however, HIVST could lengthen the time between facility visits if testing is conducted at home [14]. During the COVID‐19 pandemic, WHO recommended HIVST as a way to maintain essential services, including PrEP delivery [15]. While many countries adapted services due to the pandemic, few programmes have yet to fully integrate HIVST into standard‐of‐care digital programmes, including as part of PrEP delivery.

From November 2020, the Viet Nam Ministry of Health, supported by WHO, piloted a web‐based HIVST platform to enable populations at elevated HIV risk to: (1) access HIV testing services, including condoms, lubricants and needle exchange distribution during a period of travel restrictions; (2) support people diagnosed with HIV to link to anti‐retroviral treatment (ART); and (3) support people testing HIV negative to initiate, or maintain PrEP use. The service sought to contribute to national and UNAIDS targets of achieving 95% of people living with HIV being aware of their status, 95% of people aware of their status accessing treatment, as well as foster wider access to combination prevention for KPs, including PrEP access [16, 17].

We investigate the reach and the effectiveness of the pilot and scale‐up of one of the first web‐based HIVST programmes and further characterize the populations reached. We report on key outcomes such as linkage to ART, and initiations or continuations on PrEP, and compare these by study phase.

## 2 METHODS

### 2.1 Study type and setting

We used a mixed‐methods explanatory sequential study design. A cross‐sectional analysis of secondary programme data was conducted, followed by an explanatory qualitative analysis of satisfaction survey free‐text responses.

**Figure 1:**
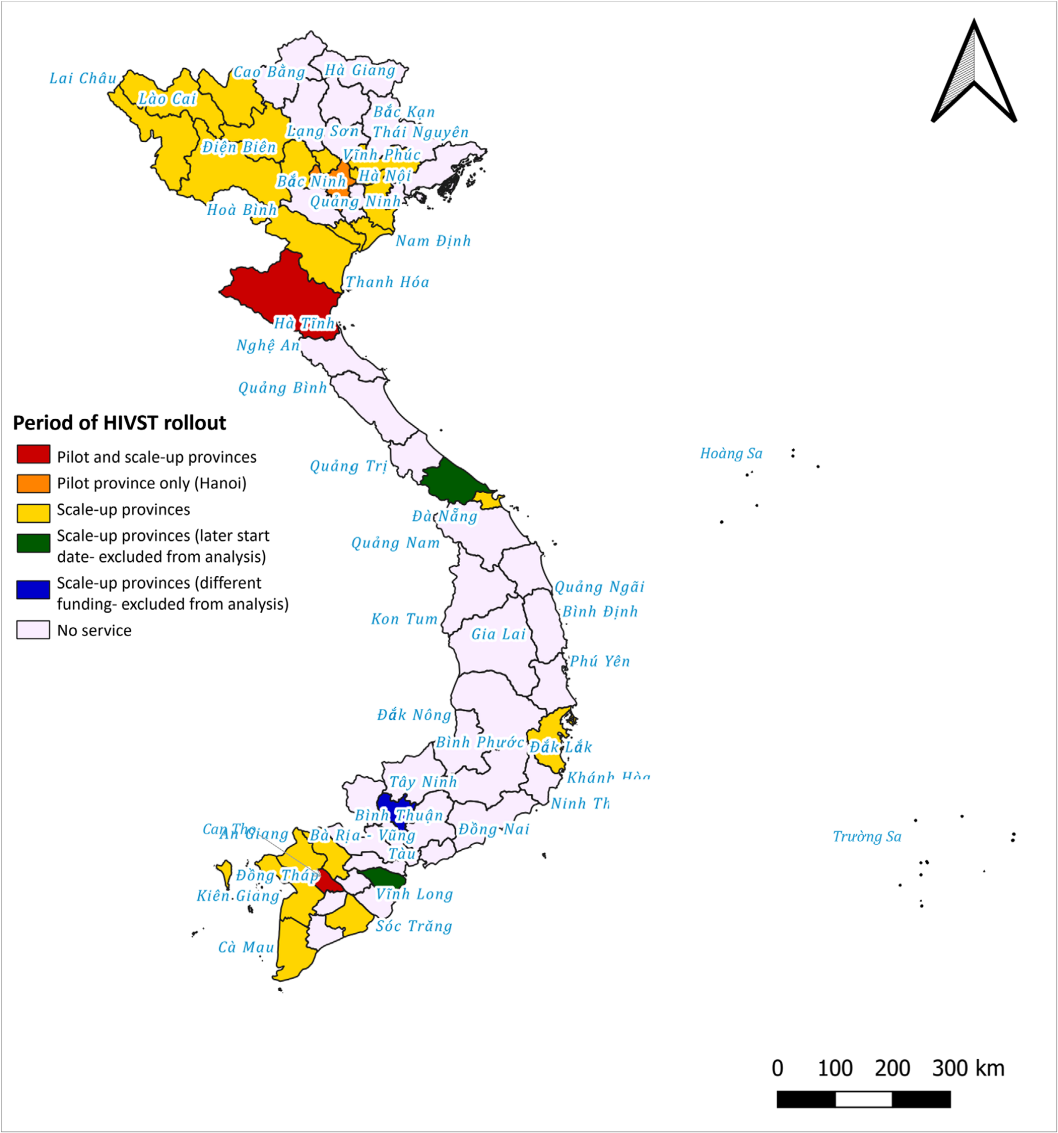
**Overview of the geographical distribution of the service rollout over time**.

The web‐based pilot initially ran in three provinces, Can Tho from November 2020 and Nghe An and Hanoi in April 2021 until December 2021. The programme ceased in Hanoi in December 2021, due to low testing numbers, but continued in Can Tho and Nghe An. In April 2022, the service was scaled‐up to full‐programmatic implementation in an additional 21 provinces. The study period and data collection dates cover November 2020–December 2022 inclusive. Provinces were selected as they are all high HIV burden provinces supported through the Global Fund (Figure 1).

### 2.2 Intervention and implementation approach

The web‐based HIVST platform used HIV oral fluid tests (OraQuick® HIV Self‐Test, Pacific Biotech Co., Ltd, Thailand). Participants created a website account, requiring eight lifetime risk‐assessment questions. If no risk factors were disclosed, participants were told that HIVST may not be relevant for them, but were still able to access the service, including for lubricants, condoms or needle exchange. After account creation, participants completed a new form to request a test kit, which included an additional risk assessment (five questions) looking at reported risk factors in the last 12 months.

During scale‐up, given the reported question burden, the registration risk assessment was dropped, retaining only the 5‐question 12‐month recall risk assessment upon test kit request. Participants could choose how to receive HIVST kits: through delivery by courier, peer educator or self‐collection. Participants were given details on the website on how to perform the self‐test kit and report their self‐test results on the online platform (although result reporting was not mandatory). After HIVST kits were successfully delivered, service staff could follow‐up via telephone call or messenger to participants who had not reported results, or those who self‐reported positive or negative results, to support further testing, PrEP or ART, as appropriate. Service linkages data were input either by service staff or participants onto the website. Participants were encouraged to complete a satisfaction survey at process end (Additional file S1 TIDieR intervention description).

#### Theory of Change

**Figure 2:**
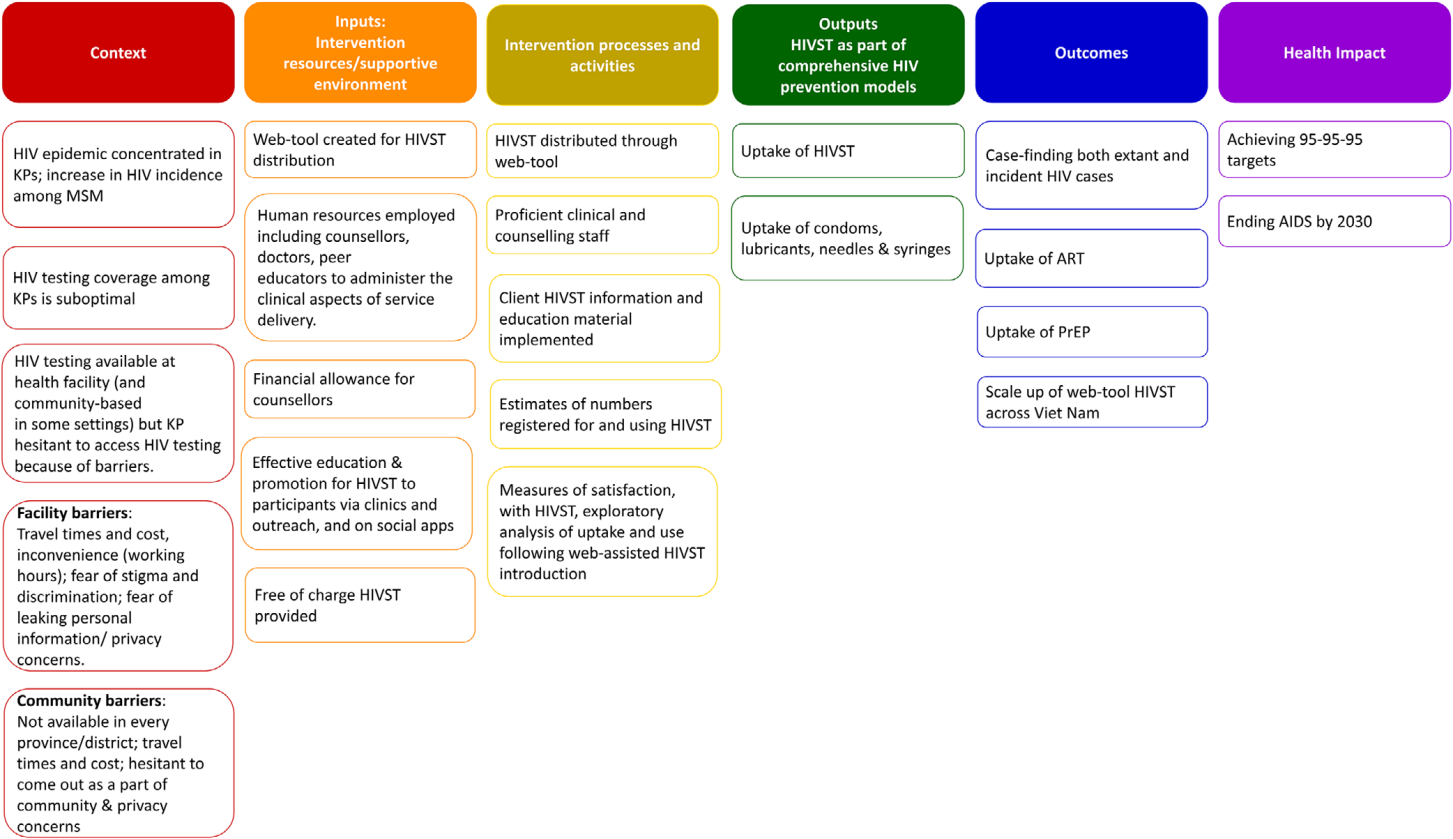
**Theory of Change of the web‐assisted HIVST programme**. Abbreviations: ART, anti‐retroviral treatment; HIV self‐testing; KP, key population; HIVST, MSM, men who have sex with men; PrEP, pre‐exposure prophylaxis.

The intervention was designed to increase uptake of HIVST and combination prevention commodities among KPs to case find incident and extant HIV, and to link people to PrEP, or ART as appropriate (Figure 2).

#### Implementation differences between provinces and from pilot to scale‐up

**Figure 3:**
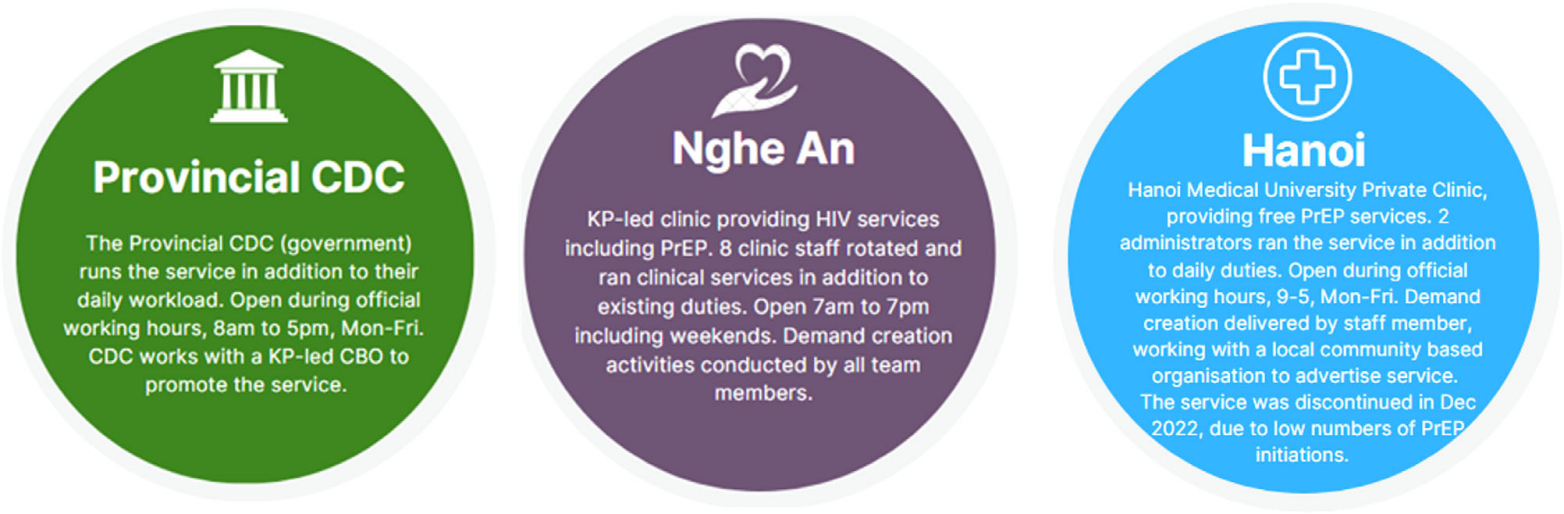
**Top‐level overview of the different service approaches between provinces**. (See Supporting information 1 for more information). Abbreviations: CBO, Community based organisation; CDC Centre for Disease Control; KP, key population; PrEP, pre‐exposure prophylaxis.

In most provinces, the service was administered through the provincial Centre for Disease Control (CDC), supported by a provincial KP network or community‐based organization (CBO). However, there were varying levels of collaboration between CBO and CDC, and various levels of service promotion by the CBO within each province, which were not captured systematically. Additionally, in Nghe An, a KP organization exclusively delivered the service, and in Hanoi, a private medical clinic delivered the service (Figure 3).

From pilot to scale‐up, the intervention package and implementation approach did not change substantially. Pilot outcomes informed scale‐up through identifying limited process barriers, which were removed at scale‐up: the registration risk assessment was dropped and the requirement to upload images of self‐test results was also dropped — otherwise, the approach and package remained consistent between phases. Additional modifications at scale‐up reflected the realities of moving to programmatic implementation; with a reduced number of staff available to support scale‐up in each province, and a reduced amount of time devoted to the activity. For example, there was less follow‐up of missing self‐test reports at scale‐up. Scale‐up was administered through the CDC working with a CBO, because of established partnerships between the Ministry of Health and CDC. In addition, during the pilot, the first 1000 people who filled out the satisfaction survey received a phone card worth ₫30,000 (≈$1.30), which was stopped at scale‐up due to budget prioritization. After the initial website was launched during the pilot in Can Tho, provincial websites were soft‐launched on social media (Additional file S1 TIDieR intervention description).

### 2.3 Analysis approach

#### Reporting guidelines

Quantitative and qualitative data were analysed separately and integrated during the results interpretation. Cross‐sectional analysis followed the STROBE checklist in conformance with guidelines for cross‐sectional studies [18]. Data were imported from MS Excel with preparation and analysis done in R and Stata 15.0 (Stata Corp, Texas, USA) [19]. Qualitative thematic content analysis followed COREQ guidelines for reporting qualitative research [20]; analysis was conducted in MS Excel using a coding framework developed by YD.

#### Ethics

Any participant registering for the web‐based service during the study timeframe was included in the analysis. An electronic informed consent form for HIVST was not required; only anonymized data were used for the purposes of programme evaluation in line with national policy. Consent was obtained for the satisfaction survey. Ethical approval was granted by the Hanoi Medical University Institution Review Board (IRB‐VN01.001/IRB00003121/FWA 00004148).

#### Exposure and outcome measurement

In alignment with the RE‐AIM framework and scientific literature [21, 22, 23], we defined intervention reach as the absolute numbers of those accessing the testing service, disaggregated by demographics, as well as the proportion of individuals conducting an HIVST of those who registered on the website. We defined effectiveness as the absolute number and proportion of individuals who reported their self‐test result, the number and proportion of those testing positive in the service, receiving confirmation testing and accessing ART, as well as the number and proportion of those initiating or maintaining PrEP use in the service.

Participant characteristics were based on self‐report from the registration form (for age and sex at birth) and the participant's risk assessment (S2). The risk assessment was informed through the CDC website, but not validated [24]. Demographic variables comprised age (grouped in‐line with national programme criteria, reflecting national age‐varying patterns of HIV‐incidence risk [25, 26] included in the descriptive analysis as a continuous and categorical variable, 15–24 years old, 25–34 years old, and 35 years and older), sex and KP group, (determined through self‐reported identity or risk behaviours either in the last 12 months or lifetime). We described multiple KP‐identity characteristics if reported. Risk factors comprised previous HIV testing (within 3 months, 3–6 months, 6–12 months or over 12 months), reported condom use, concurrent sexual partners and recent Sexually Transmitted Infection (STI) diagnosis in the last 12 months. Five ease‐of‐service use indicators, a general‐satisfaction measure and whether participants would recommend the service to a friend were drawn from an internally created self‐reported satisfaction survey, using binary category (agree/disagree) for all variables (S3).

Testing variables were based on self‐report or clinic input and described as a proportion of overall tests conducted; comprising the number of tests accessed within the service (one‐off access, two tests, three or more tests), whether a test result was reported (yes or no), HIV test result (reactive, negative or invalid), if reactive, confirmatory test accessed (yes or no), results of confirmatory test (confirmed positive, confirmed negative, unconfirmed), initiating ART if confirmed positive (yes or no). If negative, PrEP initiation or continuation (yes or no). We were not able to disaggregate between new PrEP initiations (i.e. those who started taking PrEP because the service linked them to PrEP) or PrEP continuations (i.e. participants taking PrEP prior to accessing the HIVST service), therefore, these were described together.

#### Descriptive analysis

To determine reach, we graphed flow diagrams depicting participant flow throughout the study between pilot and scale‐up and described absolute testing numbers. We summarized participant characteristics as proportions, means (standard deviation [SD]) or medians (interquartile range), as appropriate, and compared variables between pilot and scale‐up using chi‐square tests and *t*‐tests for categorical and continuous variables, respectively. We compared ease‐of‐use indicators between phases, and within demographic sub‐groups.

To determine effectiveness, we calculated the overall positive yield, compared between pilot and scale‐up as well as the case finding proportion (proportion reactive on the first test). We calculated the incidence risk of those known to be negative on their first test between pilot and scale‐up, using binomial exact confidence intervals, and compared these descriptively. We compared testing outcomes (ART initiations, and PrEP uptake or monitoring) between pilot and scale‐up.

#### Qualitative analysis

Survey responses were completed in Vietnamese and translated into English by VN. For analysis, we included free‐text participant response to the following open‐ended question: “*Do you have any suggestion to help us to improve the services? Can you put down your suggestion?*”. Responses that did not include elaboration (i.e. they did not go beyond yes or no responses) were excluded from the analysis. Thematic content analysis was conducted by YD using multiple coding cycles. We used constant comparative coding to identify areas of consensus and divergence within the pilot, and scale‐up, comparing these themes between pilot and scale‐up, provinces, age and sex assigned at birth. Final themes were agreed by YD and VN.

We triangulated the quantitative data using the qualitative analysis, then synthesized the qualitative data with the quantitative data to provide a wider explanatory context to the results.

#### Bias

Given that lifetime reported risk was dropped as part of the condensed risk assessment during scale‐up, group‐identity variables for the scale‐up are likely to underestimate KP groups compared to pilot data. The condensed risk assessment should have reduced the administrative barriers to ordering a test kit during scale‐up, therefore, potentially increasing the uptake of the intervention. The denominator for PrEP initiations or continuations was all those testing negative; it is likely not all participants would have been eligible for PrEP. We were unable to restrict the analysis to PrEP uptake in those eligible, as the risk assessment look‐back period on the web‐based platform was 12 months not 6 months, and not all national PrEP eligibility variables were collected [27]. It is expected, therefore, the reported proportion of PrEP initiations and continuations is an underestimation of uptake of those eligible for PrEP.

## 3 RESULTS

**Figure 4:**
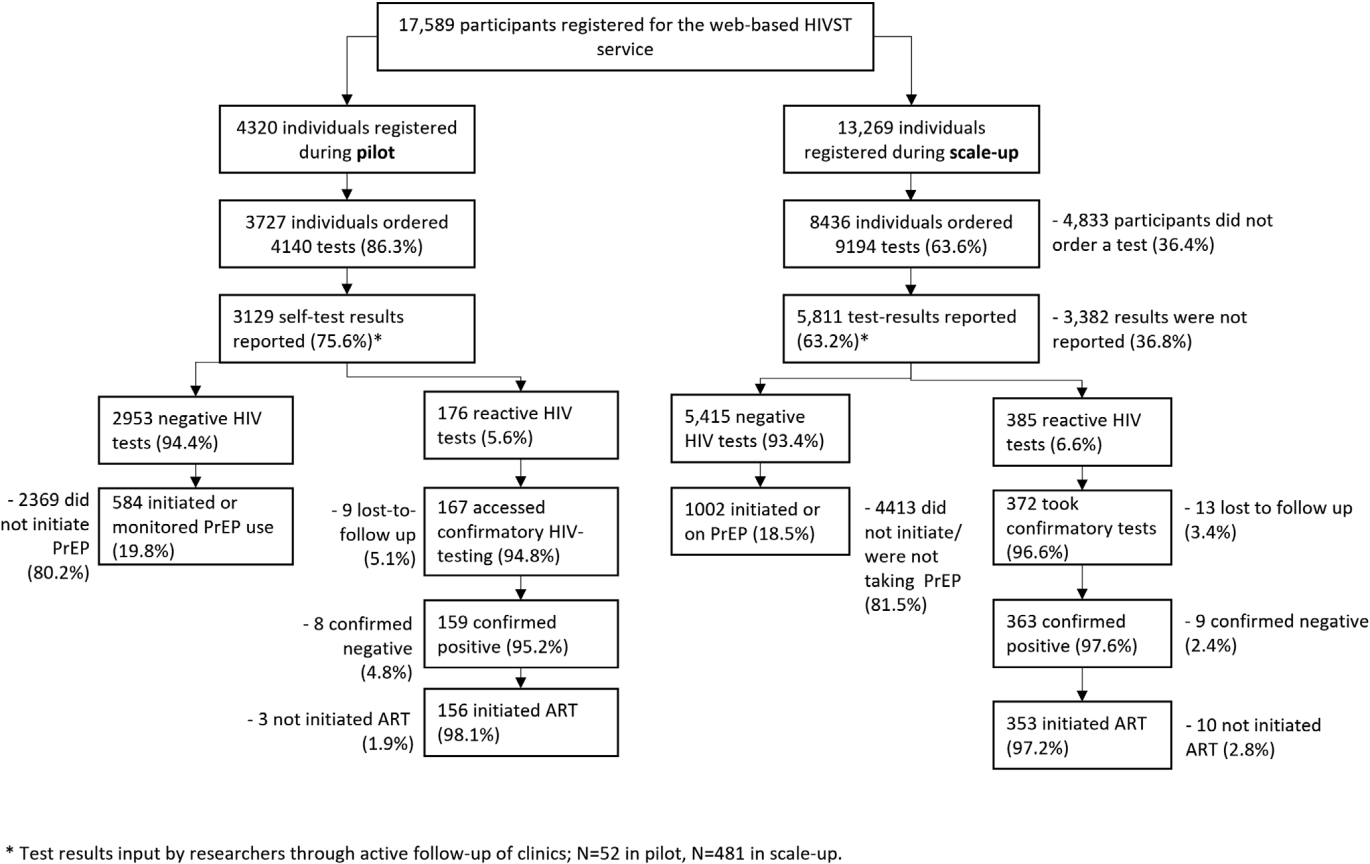
**Participant throughput by study phase**. Abbreviations: ART, anti‐retroviral treatment; HIVST, HIV self‐testing; PrEP, pre‐exposure prophylaxis.

A total of 17,589 participants registered for the web‐based HIVST service, of which 11,332 unique individuals ordered 13,334 tests (Figure 4).

**Figure 5:**
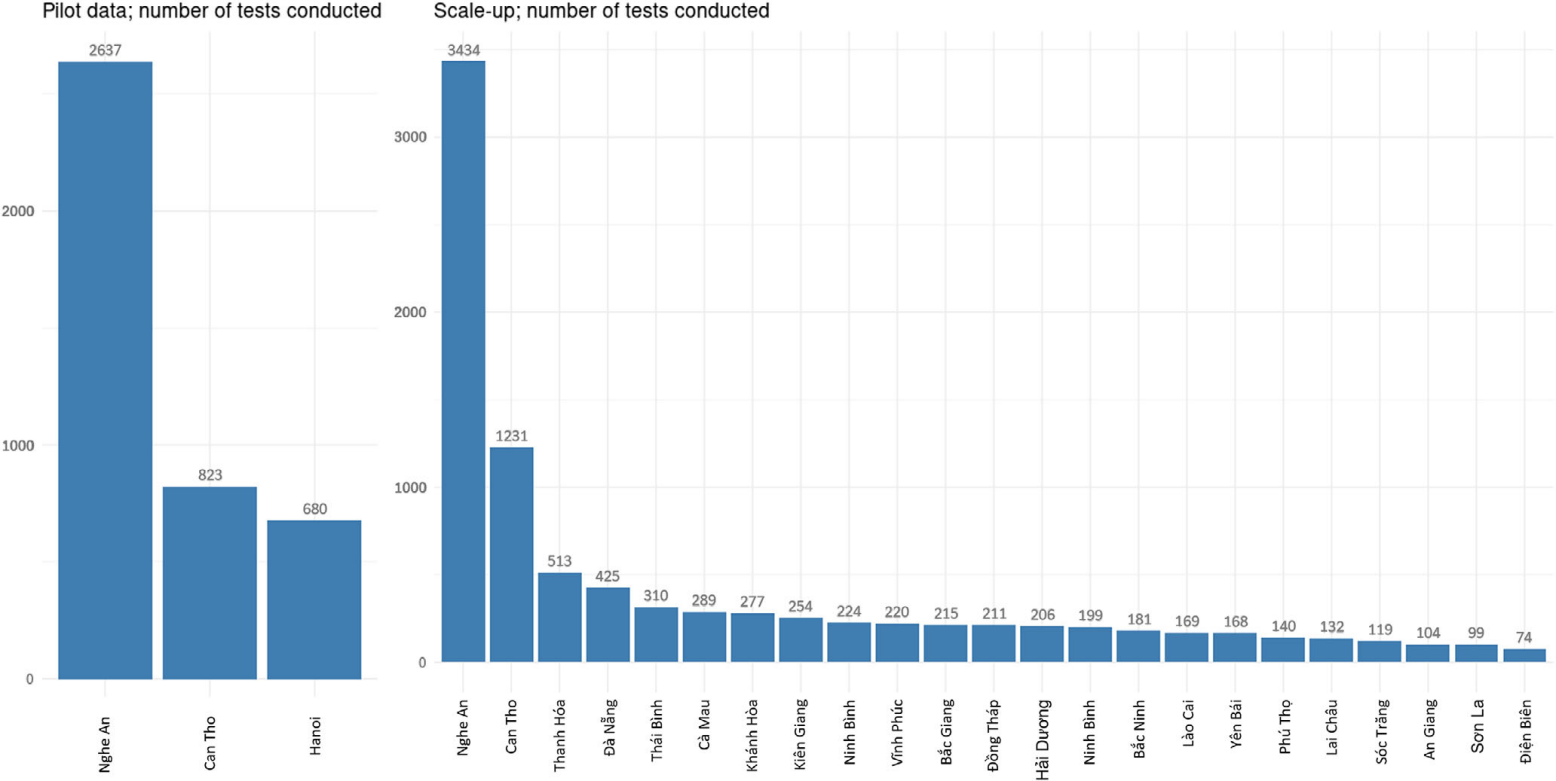
**Total number of tests conducted by province**.

3727/4320 (86.3%) individuals ordered 4140 tests in the pilot compared to 8436/13,269 (63.6%) who ordered 9194 tests during scale‐up, *p* < 0.001. This includes 831 participants who ordered HIVST kits during both the pilot and scale‐up within both time periods. In both the pilot and scale‐up, the greatest number of tests conducted was in Nghe An, where the KP‐led clinic implemented the activity, (pilot 2837/4140, 62.0%, scale‐up 3434/9194, 37.4%) followed by Can Tho. The remaining scale‐up provinces had relatively similar numbers of tests conducted (median number of test; 206) (Figure 5).

Two thousand and eighty‐six satisfaction surveys were completed after the testing intervention (1895 pilot surveys and 191 scale‐up surveys). Two hundred and sixty‐two of the 2086 satisfaction surveys also included free‐text responses eligible for thematic analysis. Almost all responses came from the pilot (234/262) with a few additional responses from the scale‐up (28/262) (S4).

There was no clear indication from thematic analysis why Nghe An reached more absolute numbers of people testing in both pilot and scale‐up, nor why there was limited numbers of people accessing the service from provinces other than Nghe An or Can Tho during scale‐up. However, a clear theme emerged with MSM pilot participants requesting the option to order on behalf of others who had confidentiality fears using the website:

“If possible, I hope the website can support registering to receive additional test kit for other (to test for a same‐sex partner or a partner who has not been tested but are afraid to order a test kit directly on the website, then I can order on their behalf), and posting the results on the website. This will help to find more positive persons and help screen other MSMs (who often rarely actively access testing services). I hope that the program will have a mechanism for this problem. Thank you.”

‐Male, 23 years old, pilot participant from Can Tho.

As a recommendation, this could act to increase the reach of the web service in terms of absolute testing numbers. However, it could also indicate that the specific delivery model in Nghe An, as administered through a KP organization, which had greater numbers of people testing, had more trust from participants in its confidentiality. While it is important to note that thematic analysis from those who used the service highlighted the service was overwhelmingly considered confidential, confidentiality concerns may have been part of varying overall reach between provinces.

**Figure 6:**
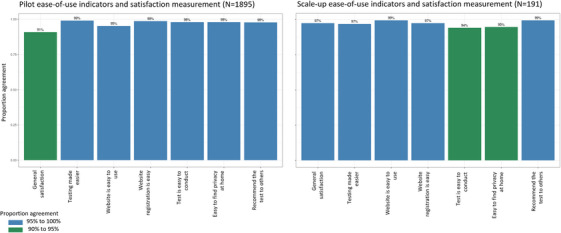
**Ease‐of‐use indicators and satisfaction measurement by implementation phase**.

The smaller proportion of initial registrations that went on to order test kits in the scale‐up compared to the pilot (86.3% compared to 63.6%) was not demonstrated by the satisfaction survey; in the scale‐up, there were decreases in reported ease of use of the test and ability to find privacy at home, but nothing indicative of why more registrations resulted in less test‐kit orders (Figure 6).

Thematic analysis did not identify any differences between phase that could explain a smaller proportion of registrations ordering test kits in the scale‐up: overall, there were high levels of reported ease of use of the test kit, the website interface and the self‐testing process from start to finish:

“Easy to order, easy to do.”

‐Male, 22 years old, pilot participant from Nghe An.

Service participants were young; over a third were under the age of 25 years old (4309/11,332, 38.0%), predominantly male (9418/11,332, 83.1%) and over half identified as MSM (6437/11,332, 56.8%). Almost half of the participants had never tested prior to accessing the service (5069/11,332, 44.9%) (Table 1).

**Table 1:** Participant characteristics by period of web‐based HIV self‐testing programme

	Total participants	Pilot participants	Scale‐up participants	
Covariates^a^	*N* = 11,332 (%)	*n* = 3727 (%)	*n* = 8436 (%)	*p*‐value^*^
**Demographics**				
Age, mean (SD)	28.8 (8.9)	26.3 (6.2)	29.7 (9.5)	<0.001^b^
15–24 years old	4309 (38.0)	1678 (45.3)	2954 (35.0)	<0.001
25–34 years old	4685 (41.3)	1710 (46.2)	3417 (40.5)	
35 years old and older	2314 (20.4)	315 (8.5)	2061 (24.4)	
Sex assigned at birth				
Female	1913 (16.9)	392 (10.5)	1595 (18.9)	<0.001
Male	9418 (83.1)	3335 (89.5)	6840 (81.1)	
MSM^†^				
No	4895 (43.2)	736 (19.7)	4147 (49.2)	<0.001
Yes	6437 (56.8)	2991 (80.3)	4289 (50.5)	
FSW				
No	10,943 (96.6)	3567 (95.7)	8139 (96.5)	0.039
Yes	389 (3.4)	160 (4.3)	297 (3.5)	
Transgender				
No	11,222 (99.0)	3674 (98.6)	8326 (98.7)	0.602
Yes	110 (1.0)	53 (1.4)	110 (1.2)	
PWID				
No	10,074 (94.8)	3513 (94.3)	7979 (94.6)	0.470
Yes	592 (5.2)	214 (5.7)	457 (5.4)	
ATS user				
No	11,093 (97.9)	3597 (96.5)	8285 (98.2)	<0.001
Yes	239 (2.1)	130 (3.5)	151 (1.8)	
**Risk factors in the past 12 months**				
Previous HIV test				
Never tested	5069 (44.9)	1897 (51.3)	3549 (42.1)	<0.001
3–6 months prior	2659 (23.5)	760 (20.5)	2132 (25.3)	
6–12 months prior	2030 (18.0)	489 (13.2)	1640 (19.5)	
More than 12 months	1542 (13.7)	555 (15.0)	1105 (13.1)	
Condom use				
Never (or rarely)	2094 (18.6)	564 (15.3)	1626 (19.3)	<0.001
Sometimes	4817 (42.9)	911 (24.7)	4055 (48.1)	
Often	4317 (38.4)	2209 (60.0)	2745 (32.6)	
Concurrent sexual partners				
No	4737 (55.4)	1598 (42.9)	3378 (59.8)	<0.001
Yes	3805 (44.5)	2129 (57.1)	2268 (40.2)	
Previous STI diagnosis				
No	8408 (89.1)	3475 (93.2)	5692 (87.0)	<0.001
Yes	1028 (10.9)	252 (6.8)	850 (13.0)	

Abbreviations: ATS user, amphetamine‐type stimulant user; FSW, female sex worker; HIVST, HIV self‐test; MSM, men who have sex with men; PWID, people who inject drugs; SD, standard deviation; STI, sexually transmitted infection.

* Chi square tests of association.

^†^ MSM breakdown described:

**Pilot**; Of 2991 MSM, 153 (5.1%) were PWID; 67 (2.2%) were ATS users.

**Scale‐up**; Of 4289 MSM, 444 (10.4%) were PWID; 147 (3.4%) were ATS users.

^a^ Missing data described:

**Pilot**; Missing: age (24/3727, 0.6%); Previous HIV test (26/3727, 0.7%); Condom use (43/3727, 1.2%).

**Scale‐up**; Missing: age (4/8436, 0.05%); Sex (1/8436, 0.01%); Previous HIV test (10/8436, 0.12%); Condom use (10/8436, 0.12%); Concurrent sexual partners (2790/8436, 33.1%); Previous STI diagnosis (1896/8436, 22.5%).

^b^ Unpaired *T*‐test, assuming equal variances.

Scale‐up participants were older (mean age: 29.7 years, SD: 9.5 compared to pilot: 26.3 years, SD: 6.2, *p* < 0.001, *p* < 0.001), with a broader range of demographics; there were almost twice as many participants assigned female at birth accessing the service in scale‐up (scale‐up; 1595/8436, 18.9% compared to pilot; 392/3727, 10.5%, *p* < 0.001). MSM made up over half of the scale‐up participants compared to almost all pilot participants (scale‐up: 4289/8436, 50.5% compared to 2991/3727, 80.3%, *p* < 0.001). There was also strong evidence of differences (*p* < 0.001) in ATS use, previous HIV testing history, sexual partner concurrency and previous STI diagnosis. However, there was no apparent risk association with these differences; scale‐up participants reported a greater proportion of previous STI diagnosis and not often using a condom, but smaller proportions of ATS use and concurrency in sexual partners. The qualitative analysis gave no indication as to why there was a broader range of demographics within the scale‐up period.

Of all tests conducted, nearly three‐quarters were first‐time tests in the service, (9855/13,334, 73.9%), with two‐thirds of test results reported (8940/13,334, 67.0%). Of these reported test results, 6.3% were reactive (561/8940). Almost all tests sent for confirmatory testing were confirmed positive (522/539, 96.8). Of these confirmed positive tests, almost all initiated ART (509/522, 97.5%). The case finding proportion, that is proportion reactive on their first test in the service, was 7.6% (95% CI: 7.0–8.3%, 494/6492); thereafter, the incidence risk of those known negative on their second test was 3.8% (95% CI: 2.9–4.9%, 58/1535) decreasing to 1.0% for those on their third or more test (95% CI: 0.5–1.9%, 9/902). Three percent (3.2%, 17/539) of HIVST were found on confirmatory testing to be false reactive (Table 2).

**Table 2:** Care and prevention cascades by phase of web‐based HIV self‐testing programme

	Aggregate testing outcomes	Pilot	Scale‐up	*p*‐value^a^
Care and prevention characteristics	*N* = 13,334 (%)	*n* = 4140 (%)	*n* = 9194 (%)	
**Number of tests within service**				
One‐off access	9855 (73.9)	2799 (67.6)	7056 (76.8)	<0.001
Two tests	2288 (17.2)	696 (16.8)	1592 (17.3)	
Three or more tests	1191 (8.9)	645 (15.5)	546 (5.9)	
**Test result report**				
No	4394 (33.0)	1011 (24.4)	3383 (36.8)	<0.001
Yes	8940 (67.0)	3129 (75.6)	5811 (63.2)	
**HIV test result**				
Negative	8379 (93.7)	2953 (94.4)	5426 (93.4)	0.063
Reactive	561 (6.3)	176 (5.6)	385 (6.6)	
*Of participant's testing reactive*				
Accessed confirmatory testing				
No	22 (3.9)	9 (5.2)	13 (3.4)	0.325
Yes	539 (96.0)	167 (94.8)	372 (96.6)	
*Of participant's accessing confirmatory test*				
Confirmatory test result^b^				
Positive	522 (96.8)	159 (95.2)	363 (97.6)	0.145
Negative	17 (3.2)	8 (4.8)	9 (2.4)	
*Of participant's confirmed positive*:				
Initiating ART				
No	13 (2.5)	3 (1.9)	10 (2.6)	0.558
Yes	509 (97.5)	156 (98.1)	353 (97.2)	
*Of participant's testing negative*:				
Initiating PrEP				
No	6777 (80.9)	2364 (80.2)	4413 (81.5)	0.124
Yes	1602 (19.1)	584 (19.8)	1002 (18.5)	
*Of all participants with reported test results*				
Initiating either PrEP or ART				
No	6845 (76.6)	2389 (76.4)	4456 (76.7)	0.724
Yes	2095 (23.4)	740 (23.6)	1355 (23.3)	

Abbreviations: ART, anti‐retroviral treatment; PrEP, pre‐exposure prophylaxis.

^a^ Chi square test of association.

^b^ Of those testing reactive and confirmed either positive or negative, accessing PrEP:

**Pilot**; Of seven participants confirmed negative, three went on to access PrEP.

**Scale‐up**; Of 10 participants confirmed negative, five went on to take or were already taking PrEP. Of 363 participants confirmed positive, one was taking PrEP.

Between the pilot and scale‐up, there was strong evidence of differences in the proportion of test results reported (pilot: 3129/4140, 75.6% compared to scale‐up: 5811/9194, 63.2%, *p* < 0.001). During the pilot, thematic analysis identified the importance of having live peer educators or staff during the self‐testing process, for example through pre‐test information at clinics, peer educators on hand to deliver test kits and clinic linkages for follow‐up. Although the theme was also remarked upon within both Can Tho and Hanoi in the pilot, and across all ages and sexes assigned at birth, this theme was particularly relevant in the pilot data from Nghe An, with many explicitly mentioning the Nghe An KP organization which administered the service by name:

“Thank you Glink clinic for giving me courage and motivation to overcome this shock.”

‐Male, 31 years old, pilot participant from Nghe An.

“Thank you Glink and the project for implementing a meaningful activity.”

‐Female, 42 years old, pilot participant from Nghe An.

“Glink's staff is attentive and enthusiastic. Friendly and confidential service.”

‐Male, 31 years old, pilot participant from Nghe An.

This may indicate that the greater self‐test report proportion in the pilot was a result of the active follow‐up provided by staff and peer educators. Within the scale‐up data, limited to primarily Can Tho province, there was no mention of staff support. When considerations were made around support, it was to call for an interactive web‐based platform, with a live‐chat function as a potential proxy for staff follow‐up:

“Can we have chat function in the web so that we can ask and get answer instantly?”

‐Male, 20 years old, scale‐up participant from Can Tho.

Changing delivery models over time with less active follow‐up as the programme expanded to implementation from the pilot may have had a role to play in the reduced proportion reporting their self‐test results in the scale‐up compared to the pilot (S1).

However, on all other testing outcomes, there were limited differences in the proportions between scale‐up and pilot; there was some evidence of a difference between the numbers testing reactive in the pilot compared to scale‐up (pilot: 176/3129, 5.6% reactive compared to scale‐up: 385/5811, 6.6% reactive, *p* = 0.063). Although the fewer test results reported in the scale‐up could have affected the estimated positivity/yield, as a crude proportion of total tests conducted, the reactive yield was comparable between pilot and scale‐up (pilot, 4.3% [176/4140], scale‐up, 4.2% [385/9194], *p* = 0.871).

Additionally, there were also no differences in terms of the proportion accessing confirmatory testing (pilot: 167/176, 94.8%, scale‐up: 372/385, 96.6%, *p* = 0.325), and proportion confirmed positive (pilot: 159/167, 95.2%, scale‐up; 363/372, 97.6%, *p* = 0.145), with almost all participants initiating ART (pilot: 156/159, 98.1%, scale‐up: 353/363, 97.2%, *p* = 0.558). It is, however, important to note in absolute terms, almost twice as many people initiated ART during scale‐up.

The web‐based self‐testing service was also designed so that participants with a negative self‐test report could manage their PrEP use, either through linking eligible participants to clinics for a PrEP initiation or through enabling PrEP users to self‐test to stay on PrEP. Almost one‐fifth of all participants with a reported negative test in both the pilot and the scale‐up initiated PrEP or were using the service to self‐test while taking PrEP (pilot; 19.8%, scale‐up; 18.5%, *p* = 0.124).

However, despite relatively high PrEP use in the pilot and scale‐up, thematic analysis indicated more demand for PrEP linkages than was afforded by the web platform with some participants requesting more active follow‐up for PrEP:

“This website should link to PrEP clinics to introduce the participants which can be convenient.”

‐Male, 21 years old, scale‐up participant from Can Tho

The PrEP theme came through exclusively from scale‐up analysis (Can Tho province); it could be, therefore, that again as the service moved from pilot to scale‐up, the less‐active delivery approach being used by staff meant there were drop‐offs in terms of active follow‐up of participants for PrEP.

We combined ART and PrEP initiations or continuations as a composite measure to investigate the proportion that engaged with any type of onward service of all those tested in the service. Overall, 2095 tests of 8940 test results reported (23.4%, 95% CI: 22.6–24.3%) resulted in ART initiation or PrEP initiation or continuation. There was no evidence of difference between pilot or scale‐up (*p* = 0.724), with 740 pilot tests of 3129 reported (23.6%; 95% CI: 22.2–25.2%) resulting in ART initiation or PrEP initiation or continuation, and 1355 of 5811 scale‐up tests (23.3%; 95% CI: 22.2–24.4%) resulting in ART initiation or PrEP initiation or continuation.

Thematic analysis identified additional recommendations for a more integrated web‐based service offer to increase service effectiveness through including both PrEP and HIV treatment with online dispensation in both the pilot and the scale‐up.

“It would be great if we can receive PrEP or HIV treatment from the web.”

‐Male, 30 years, pilot participant from Hanoi.

## 4 DISCUSSION

The pilot web‐based HIVST service reached many people who had never tested before and with ongoing HIV risk, particularly among young MSM. Lay participants testing for the first time were able and willing to use the web‐based self‐test service. Many of the effects and benefits demonstrated during the pilot phase were replicated during scale‐up illustrating how web‐based HIVST programmes can be important for reaching people with ongoing risk in need of testing, prevention and treatment options; particularly relevant as the service was considered highly confidential in a context where sexual identity is still stigmatized. As would be expected in any testing service, there were some false‐reactive HIVST which may have been interpretation issues, user error or testing in a setting with low prevalence [28]; however, as is recommended following any single/initial reactive tests, these participants engaged with confirmatory testing. While scale‐up efforts did reach more people and different groups missed during the pilot phase, such as women and older age groups, it revealed fewer orders from initial registrations, and less self‐test result reporting. Nationally, HIVST has not been used to initiate or maintain PrEP use. However, within the web‐based HIVST platform, we saw high levels of PrEP uptake and maintenance, with almost twice as many people on PrEP during the scale‐up.

There was variability between provinces demonstrating how similar programmes can achieve different outcomes, with Nghe An delivering the most test kits. This suggests that in addition to the background epidemiology, the strategies used here reached those with high ongoing HIV risk. Nghe An was unique in the service, as it was carried out by a KP‐led clinic rather than a provincial CDC. The emphasis placed on staff support and peer delivery as what made the service acceptable from the qualitative analysis was drawn primarily from the pilot in Nghe An.

The scale‐up reached more participants in absolute terms; however, the number of individuals registered and tested in the scale‐up was only three times more than the pilot. The scale‐up also had a lower test‐result report rate. These differences are not completely unexpected; as the programme broadened, there was less staff time available to invest in the service, as well as less focus on active participant follow‐up, that is more reliance on individual result reporting. This is not dissimilar to experiences in China where HIVST services with web‐based real‐time instruction, pretest and post‐test counselling have been highly effective in increasing HIVST uptake but required too many resources to implement and sustain [29]. The sustainability of the web‐based service was not assessed in this analysis. However, qualitative analysis indicates a potential role for artificial intelligence. For instance, the use of Artificial Intelligence (AI)‐assisted chatbots or the use of virtual humans to facilitate testing processes and thereby reduce the amount of staff time required to administer the service is an area of increasing research interest [29]. There is emerging experience as well on how AI can be used to generate demand for services like testing and PrEP, as well as reading and reporting self‐test results, that can also be further explored [30, 31]. Qualitative analysis also identified potential for network testing, where participants can order kits on behalf of their partners which has been demonstrated as increasing service reach to additional individuals at risk [32, 33, 34].

Despite these differences, with scale‐up the test‐result report proportion aligned with other digitally assisted HIVST programmes, including SMS‐assisted models (54–94%), as well as other scale‐up programmes in China (48–88%) [11, 35]; despite less active follow‐up of results post‐self‐test by the service administrators, there remained a comparable level of results reporting. The reported reactive proportion was also greater in scale‐up than the pilot, and there was a greater absolute number of people initiating ART. This is an important finding as HIVST generally is associated with decreases in linkage to care and ART initiations when compared to standard HIV testing services [23]. Although more active follow‐up may have elevated the level of result reporting, considering the high reactive rate, high proportion of first‐time testers and high proportion of KPs, scale‐up continued to target those at elevated risk of HIV. Our findings are similar to previous studies which have indicated that many participants self‐testing are often willing to share their results, especially when interventions are led by the community [36, 37]. Optimizing PrEP initiations and maintenance through the service also represents a novel area of research for web‐based HIVST approaches, which may generate improved data for HIVST uptake and use [35].

We were unable to evaluate intervention adoption or implementation as this was a programmatic data analysis without key informant interviews. We were unable to evaluate the maintenance of the intervention because implementation is ongoing. Maintenance assessment and costing are planned for future analyses. Other limitations result from the routine nature of the data collected. There was a considerable amount of data missing; particularly with the scale‐up 12‐month recall risk factors on concurrency in sexual partners (2790/8436, 33.1%) and previous STI diagnoses (1896/8436, 22.5%), making it difficult to determine the risk profile of these participants; the risk assessment survey was also not validated. However, this is less problematic given the high reactive yield in scale‐up indicates the service reached populations at elevated risk of HIV.

Changes in the registration process during scale‐up could have underestimated the number of KP members accessing the service; however, the increase in those assigned female at birth implies the broadening participant profile in service scale‐up was not an artefact of changes introduced by group identity reporting.

Qualitative analysis was limited; thematic analysis was drawn from one free‐text response to a satisfaction survey which was translated, of which less than 10% were eligible for analysis (with highly limited representation from the scale‐up data). Only those who completed the intervention responded to the satisfaction survey. Therefore, reasons for broadening demographics between pilot and scale‐up and fewer test requests from total registrations were unable to be addressed through qualitative analysis. It is also likely that those with fewer positive experiences were less likely to respond, thereby potentially overstating the acceptability of the intervention.

This study produced important insights on how to implement virtual interventions. With there being rapid advances in technology, including the future use of artificial intelligence together with self‐testing and self‐care in programmes, this study provides practical information for other countries seeking to scale‐up these strategies, together with strong community delivery components. Efforts to replicate the methods utilized with community delivery models in Nghe An should be explored as part of future implementation in Viet Nam and more widely. This is one of the first real‐world evaluations of programme scale‐up that indicates web‐assisted HIVST may also be able to increase PrEP uptake and the total number of people taking PrEP. Further research is needed in operational settings to optimize HIVST‐PrEP and prevention more broadly, including to meet a clear demand for both social network testing and direct linkage into PrEP initiations.

## 5 CONCLUSIONS

Web‐based HIVST in Viet Nam can reach people, including those who have never sought HIV testing before, at elevated risk of HIV, identifying more people with HIV, in terms of both case finding and incident HIV cases, and facilitate uptake of both ART and PrEP. Such programmes provide a critical way to expand testing services and can contribute to achieving global prevention and treatment targets to eliminate HIV as a public health threat by 2030. Efforts are now needed to expand the programme and optimized implementation to ensure it is person‐centred and adapts to the needs of users and is targeted effectively and serve users. The programme is actively working on fully costing the service and enabling self‐test kit orders for the friends or partners of service users. Finally, the service aims to improve the data capture tools to accurately monitor PrEP pathways for self‐testers to better understand the service's impact on PrEP initiations.

## COMPETING INTERESTS

All authors have no competing interests to declare.

## AUTHORS’ CONTRIBUTIONS

VTTN, VHS and CJ developed the protocol. VTTN, VHS, PTTH, PDM, TMT, HMT, DTG, LTT and VDH supported implementation. YD and ATC conducted and finalized data analysis, with support from VTTN, CJ and RB. YD drafted the manuscript with support from ATC and VTTN. VTTN, CJ, ATC and RB provided critical feedbacks for finalization of the manuscripts. All other authors including PTTH, VHS, PDM, TMT, HMT, DTG, LTT and VDH reviewed and provided comments on the manuscript.

## FUNDING

This research was funded through UNITAID, UNAIDS and Global Fund:

- UNITAID funding reference: UNITAID‐WHO HIV and Co‐Infections/Co‐Morbidities Enabler Grant (HIV&COIMS) led by Cheryl Johnson
- UNAIDS Unified Budget, Results and Accountability Framework (UBRAF) Country Envelop
- Global Fund to Fight AIDS, Tuberculosis and Malaria funding: Global Fund HIV project in Viet Nam

## DISCLAIMER

The views expressed in this manuscript are those of the authors and do not necessarily represent the official position of the WHO.

## CME STATEMENT

This article is published as part of a supplement supported by unrestricted educational grant by ViiV Healthcare.

Credits Available for this Activity: American Medical Association (AMA Credit).

Washington University School of Medicine in St. Louis designates this enduring material for a maximum of 1 AMA PRA Category 1 Credit™. Physicians should claim only the credit commensurate with the extent of their participation in the activity.

## Supporting information


**Additional file 1**. TIDieR description of the web‐based HIV self‐test distribution intervention

**Additional file 2**: Summary of risk assessment questionnaires for self‐administration

**Additional file 3**: Survey Questionnaires

**Table S1**: Participant demographics of respondents to qualitative analysis

**Figure S1**: Web‐Assisted HIVST procedure

## Data Availability

The data that support the findings of this study are available on request from the corresponding author. The data are not publicly available due to privacy or ethical restrictions.
