# Supplementary material for: Investigating the effectiveness of web‐based HIV self‐test distribution and linkage to HIV treatment and PrEP among groups at elevated risk of HIV in Viet Nam provinces: a mixed‐methods analysis of implementation from pilot to scale‐up
Source: J Int AIDS Soc. 2024 Jul 5;27(Suppl 1):e26264. doi: 10.1002/jia2.26264 (PMC11967693; doi:10.1002/jia2.26264)
Supplement: Supplementary file 1 — Additional file 1. TIDieR description of the web‐based HIV self‐test distribution intervention [file JIA2-27-e26264-s002.docx]

**Appendix 1. TIDieR description of the web-based HIV self-test distribution intervention.** *Stratified by phase and province. Content highlights differences between provinces and across intervention phase.*

| **Intervention Characteristics****†** | **Pilot period** | | | **Scale up period** | |
| --- | --- | --- | --- | --- | --- |
|  | Can Tho | Hanoi | Nghe An | Nghe An | Can Tho and an additional 21 provinces, also administered through the provincial CDC* |
| Name of the intervention | Web-based HIV self-testing. | | | | |
| **Why:**  Rationale for the intervention (Theory of Change) | Key populations experience known barriers accessing existing HIV services in Viet Nam, because of stigma, discrimination, limited availability of services, and structural challenges such as criminalisation of sex work and injecting drug use. The provision of a web-based HIV self-testing service including associated human resources, education, and awareness campaigns and free of charge HIV self-tests aimed to increase uptake of HIV self-testing, and condom, lubricants, and needle services. Anticipated outcomes were case-finding of incident and extant HIV cases, as well as uptake of ART, PrEP and service scale-up across provinces. The health impact was to contribute to 95-95-95 targets (with 95% of people in Viet Nam aware of their HIV status, and 95% accessing ART, as well as to end AIDS by 2030. | | | | |
| **What:**  *Materials* | *Demand creation:*  Across all implemented provinces and across both phases, posters with slogans to self-test for HIV and QR codes with links to the website were distributed; in locations informed through the intelligence of the community-based organisation or key population network working alongside the provincial CDC or, in Hanoi and Nghe An, through the different delivery agencies (see “**Who**”). An example of these posters is included below.  Social media was also used to promote the intervention (Facebook, BlueD, Zalo, and Tiktok,), as well as livestreams on key population community member social media pages. The website that housed the testing intervention was developed by an external IT partner. The content of the website was informed through WHO and Viet Nam Ministry of Health. Regardless of province, the website had the same landing page. The website was only formally launched in Can Tho.  *Testing-materials*  There was no difference in the material offer from the intervention. Clients could order any of the following commodities using the website URL:  HIVST, condoms, lubricants, 1ml and 2ml Syringes and Needles. 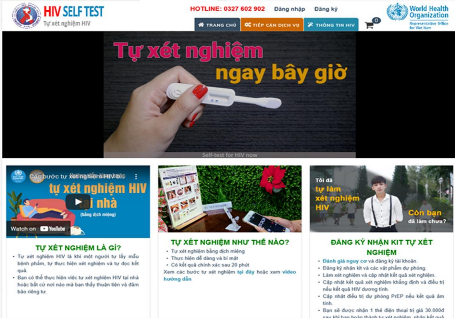 Example website landing-page 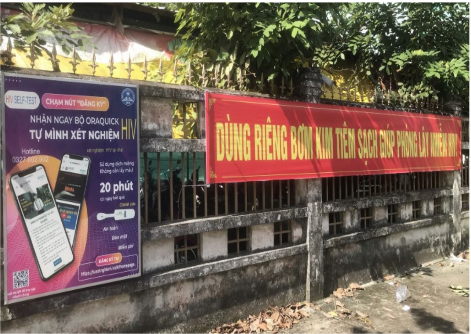 Example service poster 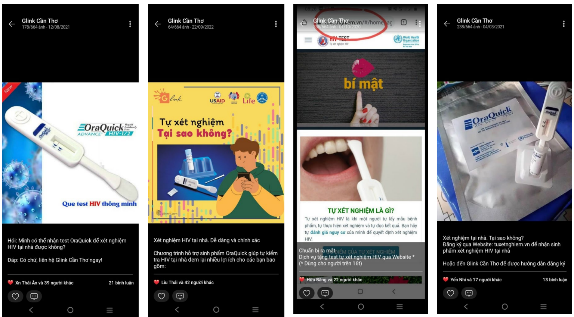 Example social media campaign | | | | |
| **What**:  *Procedures* | The test order procedure was relatively standardised between pilot and scale-up:  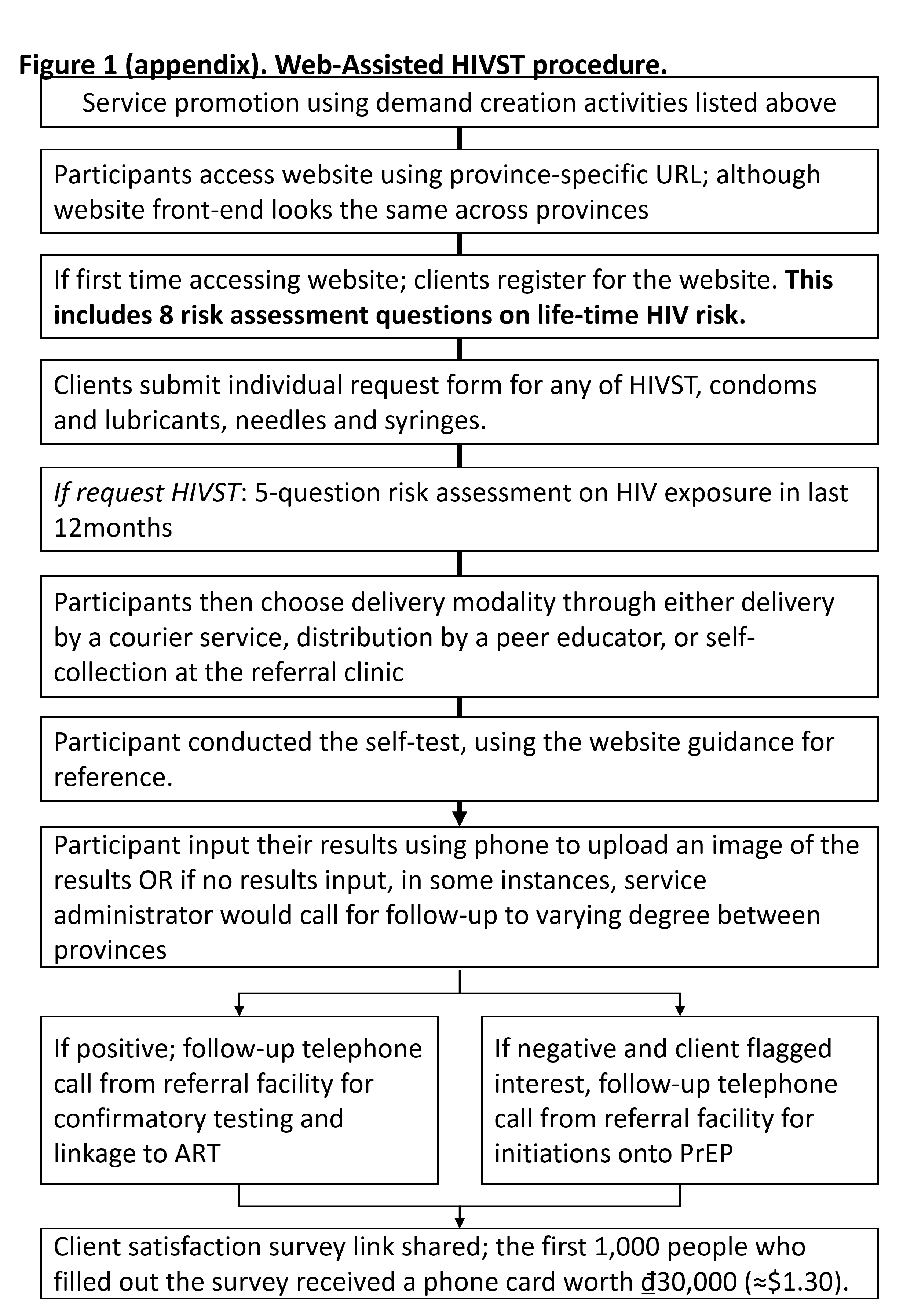 | | | There were however differences in scale-up with:   - Due to reported questionnaire burden and duplication with the HIVST kit request form, the 8-question lifetime risk assessment at registration was dropped, and only the 5-question assessment retain at kit order. - Due to reported challenges uploading images of self-test results, the requirement was dropped at scale-up. - The amount of follow-up of missing self-test reports by service administrators appeared to reduce. In the pilot, 1.7% (52/3129) results were missing that required active follow-up by the researchers to the clinics, because the client had been indicated by the facility as taking ART or PrEP, but no test result was registered on the system. With the scale-up, this increased to 8.3%, (481/5811). - There was no longer a phone-card reimbursement for completion of the satisfaction survey in the scale-up. | |
| **Who** **provided:** | In Can Tho, the provincial CDC administered the service in addition to their daily workload. The provincial CDC worked with a community-based organisation (CBO) – Glink, the sister organisation to the Glink in Nghe An - to promote the service.  The CDC conducted participant follow-up and could also provide confirmatory testing and PrEP services. Additional services, including ART, was through referral facilities (e.g., public, and private PrEP clinics, provincial and district confirmatory laboratories, provincial and district hospital providing ART services). In Viet Nam, ART is only provided for free at the public clinics.  Staffing complement:  *Administrative*  1 administrator managed the service including client follow-up.  *Clinical staff*  **Counsellors:** Trained on HIV testing and counselling  **Technician**: Trained on HIV testing  **Doctor**: Trained to initiate HIV treatment. | Hanoi Medical University Private Clinic was a PrEP clinic that also administered the HIVST service; i.e., the service administrator was the same facility that conducted the clinical care.  2 administrators ran the service in addition to their daily duties. Demand creation activities were conducted by a local CBO. The service was not taken through to scale-up because of low numbers of PrEP initiations.  *Clinical (internal staff)*  **Counsellors:** Trained on HIV testing and counselling  **Technician**: Trained on HIV testing  **Doctor**: Trained to initiate HIV treatment. | In Nghe An, Glink clinic administered the web service, conducted the demand creation activities, and provided free PrEP services. This is a key-population led service.  Referrals were made for confirmatory testing and for ART initiations.  8 clinical staff rotated service administration in addition to their existing duties.  *Clinical (internal staff)*  **Counsellors:** Trained on HIV testing and counselling  **Technician**: Trained on HIV testing  **Doctor**: Trained to initiate HIV treatment. | No change in staffing or service delivery model during scale-up with Nghe An.  The service in Hanoi was dropped after the pilot. | The Can Tho model was adopted for all scale-up provinces, including the same staffing and capacity at provincial level. However, there were varying levels of collaboration between the provincial CDC and the CBOs to promote the service; these differences were not reported systematically. |
| **How:**  *Mode of delivery; individual or group:* | Demand creation and service promotion happened primarily 1:1 online due to COVID19 restrictions.  The web-service was accessed by the participant.  Follow-up was through telephone, or SMS message based. | | | As COVID19 restrictions lifted, more demand creation events happened offline organized by the KP-led clinic administering the service; both group and individual awareness raising. | Similarly, as COVID19 restrictions lifted, demand creation activities were also organized offline by the CBO’s partnered with CDC in each province. |
| **Where:** | Can Tho was selected to represent the Southern part of Viet Nam as Can Tho is the centre of Mekong River delta with a high density population of MSM.  Additional detail on service promotion activities is lacking. | Hanoi was selected as a site for Northern Viet Nam. | Nghe An was selected to represent Central Viet Nam. | Scale-up was continued in Nghe An due to its high performance. | CDC provinces were selected as they had high burden of HIV and are supported by the Global Fund project. |
| **When and how much:** | There are no restrictions on what time of day a participant can order a test kit. However, a participant could order only one test kit every three months unless their test results were positive.  For any participant follow-up, the CDC administering the service worked between 8am to 5pm Monday to Fridays. This includes for test-kit deliveries. | The service was administered and clinical care available from 9am to 5pm, Monday to Friday. | The service was administered and clinical care available between 7am to 7pm including on weekends. | No change. | As Can Tho in pilot; the service worked between 8am to 5pm Monday to Fridays. |
| **Tailoring:** | The same intervention was offered to all participants, but they could request what commodities they needed. Participants could request HIVST with or without condom, lubricants, or needle syringes. | | | | |
| **Modifications:** | There were minor modifications made after the pilot to simplify the process of HIVST requesting:   - Removal of the lifetime risk screening questionnaire at registration. - Requirement for an email address was removed. - The requirement to upload an image of test results was removed. - During COVID-19 the promotion of activities was mostly online and the delivery of HIVST was interrupted because of travel restrictions. | | | - All the modifications were applied for scale-up. - There were no longer travel restrictions associated with COVID-19 and offline service promotion activities could take place. - Survey completion no longer received an airtime reimbursement. | |

† Not represented in the table is how well (planned and actual) the intervention was adhered to (fidelity), because there was no formal assessment of this.

* The delivery model in the scale-up provinces adhered to the same delivery model administered in Can Tho (i.e., the web-based service was administered through the provincial CDC), therefore these provinces have been grouped together. The provinces are: An Giang, Bắc Giang, Bắc Ninh, Cà Mau, Đà Nẵng, Điện Biên, Đồng Tháp, Hải Dương, Khánh Hòa, Kiên Giang, Lai Châu, Lào Cai, Nam Định, Ninh Bình, Phú Thọ, Sóc Trăng, Sơn La, Thái Bình, Thanh Hóa, Vĩnh Phúc and Yên Bái.
