# Supplementary material for: Investigating the effectiveness of web‐based HIV self‐test distribution and linkage to HIV treatment and PrEP among groups at elevated risk of HIV in Viet Nam provinces: a mixed‐methods analysis of implementation from pilot to scale‐up
Source: J Int AIDS Soc. 2024 Jul 5;27(Suppl 1):e26264. doi: 10.1002/jia2.26264 (PMC11967693; doi:10.1002/jia2.26264)
Supplement: Supplementary file 2 — Additional file 2: Summary of risk assessment questionnaires for self‐administration [file JIA2-27-e26264-s004.docx]

**S2: Summary of risk assessment questionnaires for self-administration**

**Q1: Which the group below you think you are belong to?** *You can select more than one option if relevant*

- Men who have sex with men
- Transgender person
- People who inject drugs
- People who use synthetic drug
- Female sex workers
- Other, please specify …………………

**Q2**. When was your last HIV test?

- Never tested before
- Less than 6 months
- 6-12 month
- More than 12 months

**Q3.** In the last 12 months, did you have any risk factors below? *(You can select more than one if relevant).*

- Having anal sex
- Having sex with two partners or more
- Diagnosed or treated with any of sexually transmitted infections (gonorrhea, syphilis, genital wart etc.)
- Having sex to exchange for money or material
- Having sex with female sex worker
- Using synthetic drugs
- Sharing needles/syringes with injecting partners
- Having sex with people living with HIV

**Q4.** In the last 12 months, how often did you use condom when having sex?

- Never used
- Sometime used
- Often used

**Q5.** In the last 12 month, did you have sex with a partner who you did not know about his/her sexual behaviors?

- Yes
- No
- Don’t remember/Don’t know
