# Supplementary material for: Investigating the effectiveness of web‐based HIV self‐test distribution and linkage to HIV treatment and PrEP among groups at elevated risk of HIV in Viet Nam provinces: a mixed‐methods analysis of implementation from pilot to scale‐up
Source: J Int AIDS Soc. 2024 Jul 5;27(Suppl 1):e26264. doi: 10.1002/jia2.26264 (PMC11967693; doi:10.1002/jia2.26264)
Supplement: Supplementary file 3 — Additional file 3: Survey Questionnaires [file JIA2-27-e26264-s003.docx]

**S3: SURVEY QUESTIONNAIRES**

**YOUR EXPERIENCES IN USING WEB-BASED HIV SELF-TESTING SERVICES**

*You are receiving this questionnaire because we understand you requested for and used HIV self-test kits from our website. We would like to know your experiences in using this online service. Your information will help us to improve the implementation of this innovative approach for further scale-up and to serve your community better in the coming time. Your personal information will not be collected for this purpose and your responses to the questionnaire will be used in group and may be presented in a report, meeting or publication paper.
By accepting and responding to this questionnaire, you have agreed that you understand and voluntarily participated in this survey. While you will not have any direct benefit from participated in his survey, you will be receiving a phone card worth VND 30,000 for your time after you complete the survey.*

**1. How would you rate your satisfaction with receiving HIV self-test service from our website ***

1 2 3 4 5

very satisfied satisfied neutral unsatisfied very unsatisfied

**2. Do you agree with the following sentence? Please mark the answer to the level of your agreement**

Requesting HIV self-test kit from the website make it much easier for me to get my HIV test *

1 2 3 4 5

strongly agree agree neutral disagree strongly disagree

**3. Is it easy for you to access the website? ***

🞏 Yes

🞏 No, please specify the reason: ………………………………………………………………………………………………………………………..

**4. Is this easy for you to create and log-in to your account in the website?**

🞏 Yes

🞏 No, please specify the reason: ………………………………………………………………………………………………………………………..

**5. How long did you receive the test kit since your request is submitted?**

🞏 1 day

🞏 2 days

🞏 3 days

🞏 > 3 days

**6. How your test kit was delivered?**

🞏 I picked-up myself

🞏 Peer educator delivered

🞏 Post office

**7. Is this easier to find a place to perform your test**

🞏 Yes

🞏 No

🞏 Other

**8. How do you rate the level of easiness or difficulty of performing your self-test**

1 2 3 4 5

very easy easy neutral difficult very difficult

**9. Which part of the testing process you have difficulty**

🞏 None

🞏 Taking oral fluid sample

🞏 Reading the result

🞏 Other, please specify ………………………………………………………………………………………………………………………………….

**9. Did you or will you recommend HIVST to your partner, family or friends?**

🞏 Yes

🞏 No

**10. Overall, are you happy with the services you received?**

1 2 3 4 5

very happy happy neutral unhappy very unhappy

**11. Would you like to recommend on how we can improve the web-based HIVST distribution services? Please write it down your suggestions**

……………………………………………………………………………………………………………………………………………………………………..

……………………………………………………………………………………………………………………………………………………………………...

……………………………………………………………………………………………………………………………………………………………………..

……………………………………………………………………………………………………………………………………………………………………...

……………………………………………………………………………………………………………………………………………………………………..

……………………………………………………………………………………………………………………………………………………………………...
