# Supplementary material for: Investigating the effectiveness of web‐based HIV self‐test distribution and linkage to HIV treatment and PrEP among groups at elevated risk of HIV in Viet Nam provinces: a mixed‐methods analysis of implementation from pilot to scale‐up
Source: J Int AIDS Soc. 2024 Jul 5;27(Suppl 1):e26264. doi: 10.1002/jia2.26264 (PMC11967693; doi:10.1002/jia2.26264)
Supplement: Supplementary file 4 — Table S1: Participant demographics of respondents to qualitative analysis [file JIA2-27-e26264-s001.docx]

**Supplement 4:**

Thematic analysis participants (N=262) were mostly assigned male at birth, young (<34 years old) and from either Can Tho or Nghe An provinces. In the pilot, differences by sex assigned at birth could be explored, as well as age differences in themes across pilot and scale-up. Within the pilot there were also sufficient responses to identify thematic differences by province (Nghe An, Can Tho and Hanoi), however Can Tho province formed most responses from the scale-up, therefore scale-up analysis was limited to age. (Table 1).

**Table 1: Participant demographics of respondents to qualitative analysis:**

| **Participant Characteristics** | **Total**  **N=262 (%)** | **Pilot**  **N=234 (%)** | **Scale-up**  **N=28 (%)** |
| --- | --- | --- | --- |
| **Sex assigned at birth**  Female | 35 (13.4) | 34 (14.5) | 1 (3.6) |
| Male | 227 (86.6) | 200 (85.5) | 27 (96.4) |
| **Age**  15 to 24 years old | 128 (48.9) | 108 (46.2) | 20 (71.4) |
| 25 to 34 years old | 112 (42.7) | 105 (44.9) | 7 (25.0) |
| 35 years old and older | 22 (8.4) | 21 (9.0) | 1 (3.6) |
| **Provinces**  Can Tho | 77 (29.4) | 55 (23.5) | 22 (78.6) |
| Hanoi | 31 (11.8) | 31 (13.2) | 0 (0.0) |
| Nghe An | 150 (57.3) | 148 (63.2) | 2 (7.1) |
| *Scale-up additional: Ca Mau, Da Nang, Hai Duong, Soc Trang* | 4 (1.5) | 0 (0.0) | 4 (14.3) |
