# Supplementary figures and images for: Investigating the effectiveness of web‐based HIV self‐test distribution and linkage to HIV treatment and PrEP among groups at elevated risk of HIV in Viet Nam provinces: a mixed‐methods analysis of implementation from pilot to scale‐up
Source: J Int AIDS Soc. 2024 Jul 5;27(Suppl 1):e26264. doi: 10.1002/jia2.26264 (PMC11967693; doi:10.1002/jia2.26264)

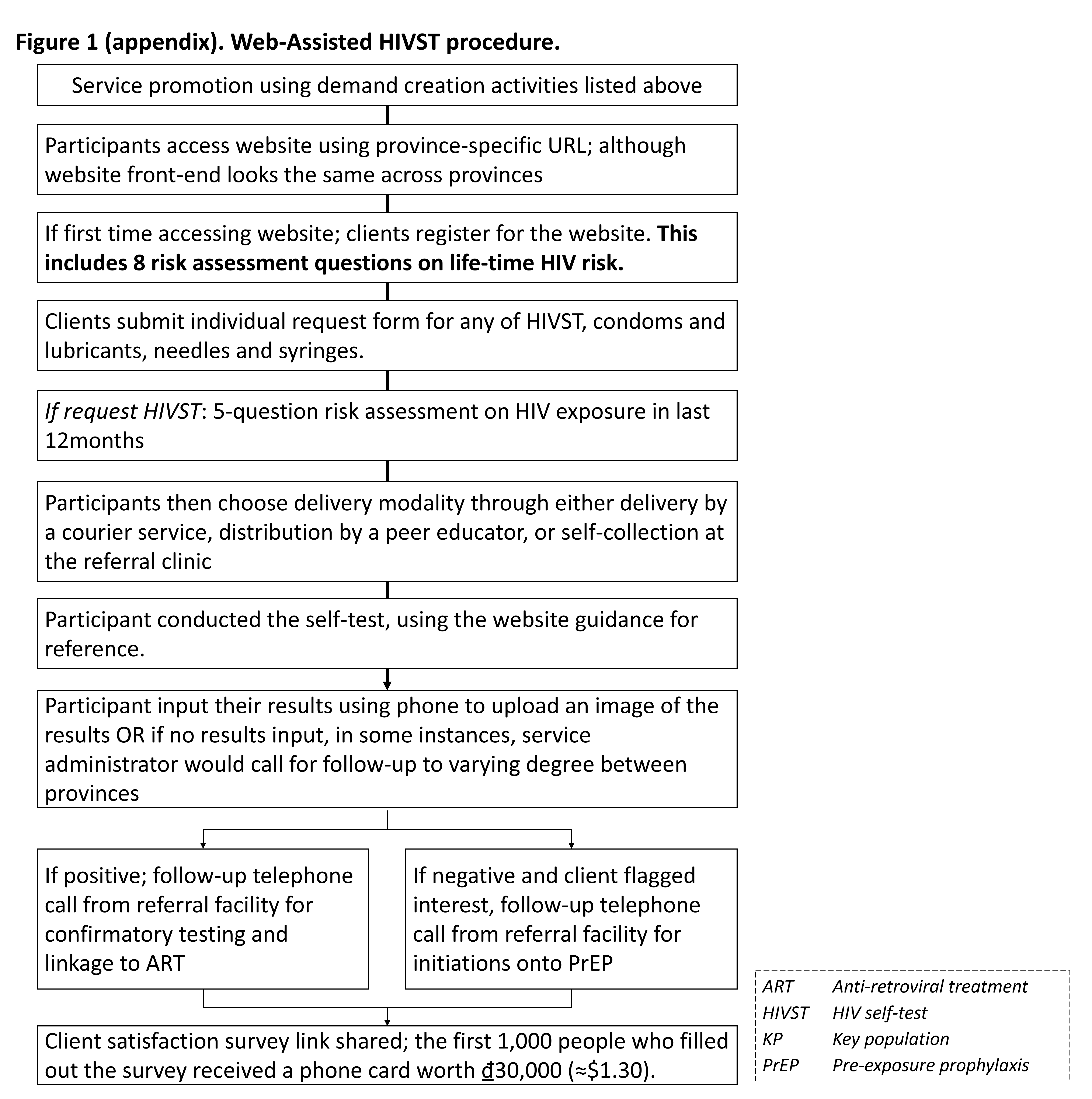

Supplement: Supplementary file 5 — Figure S1: Web‐Assisted HIVST procedure [file JIA2-27-e26264-s005.png]
